# Antidepressant effects of crocin and its effects on transcript and protein levels of CREB, BDNF, and VGF in rat hippocampus

**DOI:** 10.1186/2008-2231-22-16

**Published:** 2014-01-08

**Authors:** Faezeh Vahdati Hassani, Vahideh Naseri, Bibi Marjan Razavi, Soghra Mehri, Khalil Abnous, Hossein Hosseinzadeh

**Affiliations:** 1 School of Pharmacy, Mashhad University of Medical Sciences, Mashhad, Iran; 2 Targeted Drug Delivery Research Centre, Department of Pharmacodynamics and Toxicology, School of Pharmacy, Mashhad University of Medical Sciences, Mashhad, Iran; 3 Pharmaceutical Research Center, Department of Pharmacodynamics and Toxicology, School of Pharmacy, Mashhad University of Medical Sciences, Mashhad, Iran; 4 Pharmaceutical Research Center, Department of Medicinal Chemistry and Department of Biotechnology, Mashhad University of Medical Sciences, Mashhad, Iran

**Keywords:** Crocin, Antidepressant, Forced swimming test, qRT-PCR, Western blot

## Abstract

**Background:**

Antidepressants have been shown to affect levels of brain-derived neurotrophic factor (BDNF) and VGF (non-acronymic) whose transcriptions are dependent on cAMP response element binding protein (CREB) in long term treatment. The aim of this study was to verify the subacute antidepressant effects of crocin, an active constituent of saffron (*Crocus sativus* L.), and its effects on CREB, BDNF, and VGF proteins, transcript levels and amount of active, phosphorylated CREB (P-CREB) protein in rat hippocampus.

**Methods:**

Crocin (12.5, 25, and 50 mg/kg), imipramine (10 mg/kg; positive control) and saline (1 mL/kg; neutral control) were administered intraperitoneally (IP) to male Wistar rats for 21 days. The antidepressant effects were studied using the forced swimming test (FST) on day 21 after injection. Protein expression and transcript levels of genes in the rat hippocampus were evaluated using western blot and quantitative reverse transcription-polymerase chain reaction (qRT-PCR), respectively.

**Results:**

Crocin significantly reduced the immobility time in the FST. Western blot analysis showed that 25 and 50 mg/kg of crocin increased the levels of CREB and BDNF significantly and dose dependently. All doses of crocin increased the VGF levels in a dose-dependent manner. Levels of p-CREB increased significantly by 50 mg/kg dose of crocin. Only 12.5 mg/kg crocin could significantly increase the transcript levels of BDNF. No changes in CREB and VGF transcript levels were observed in all groups.

**Conclusions:**

These results suggest that crocin has antidepressant-like action by increasing CREB, BDNF and VGF levels in hippocampus.

## Background

Depression, a serious and prevalent mental disorder, has been predicted to be one of 10 leading causes of disabilities that affects up to 21% of world population by 2020 [1]. Due to side effects including inability to drive a car, dry mouth, constipation, and sexual dysfunction, the majority of patients have low compliance and refuse to take synthetic antidepressants in appropriate doses [2,3].Thus, there is a need for more tolerable and less toxic agents such as natural plant products which are important sources of new antidepressant drugs [4,5]. Contrary to what is expected, existing antidepressant drug treatments which based on the monoamine hypothesis are just effective in almost one-third of depressed patients. Moreover, clinical manifestations take 3–4 weeks to start; although changes in synaptic monoamine levels occur within hours. This delay in clinical efficacy may be due to neurobiological adaptive mechanisms in hippocampus including alterations in synaptic plasticity and neurogenesis which require synthesis of new proteins [6-10]. CREB (cAMP response element binding protein) is a transcription factor upregulated and phosphorylated by chronic antidepressant treatment. Phosphorylation promotes the association of CREB with CREB-binding protein, a co-activator protein that plays role in assembly of an active transcription complex, enabling target gene expression [11]. VGF (non-acronymic) and BDNF (brain-derived neurotrophic factor) whose transcriptions are dependent on CREB, involved in depressive disorders. VGF is a neuropeptide which enhances hippocampal synaptic plasticity and has roles in energy balance and regulation of homeostasis. It also acts as antidepressant-like agent in the forced swimming test (FST) behavioral model of depression [7]. BDNF, widely expressed in mammalian brain, has been implicated in survival of neurons during hippocampal development, neural regeneration, synaptic transmission, synaptic plasticity, and neurogenesis [12].

*Crocus sativus* L. (Iridaceae) stigma commonly known as saffron is widely cultivated in Iran and is used in modern and traditional medicines. In addition, results of different studies on pharmacological properties of saffron and its constituents, crocetin, crocin and safranal, are similar to findings as described by Avicenna. Crocin (crocetin digentiobiose ester), a unique water-soluble carotenoid, is one of the pharmacological active constituent of saffron [13,14]. Extensive studies has evaluated saffron extracts and crocin for their pharmacological benefits such as anti-tumor and cytotoxic [15-19], antioxidant [20], antinociceptive and anti-inflammatory [21,22], aphrodisiac [23], antitussive [24], cardioprotective and hypotensive [25-27] activities. Their various effects on central nervous system including improvement of spatial cognitive abilities [28,29], anti-anxiety action [30], reducing morphine withdrawal, morphine-induced conditioned place preference, and dependence [31,32], and anticonvulsant activities [33] were also investigated. The antidepressant effects of different extracts of stigmas, petals, and corms of *C. sativus* L. and their active constituents were evaluated in acute preclinical studies and shown to be significantly more beneficial than placebo [34-37]. In the present study, we first investigated the antidepressant effects of crocin in rats using the FST; then, the protein and transcript levels of CREB, BDNF, and VGF in rat hippocampus were measured in order to understand the underlying molecular mechanism of antidepressant effects of crocin.

## Methods

### Animals

Adult male Wistar Albino rats, weighing 250–300 g, were provided by Animal House, School of Pharmacy, Mashhad University of Medical Sciences, Iran. Four rats were housed in standard plastic cages in the colony room under 12-h light/dark cycle, 22 ± 2°C and 40-50% humidity conditions. Animals had free access to food and water before and during the study. This study was approved by the ethical committee (No:88587) of Mashhad University of Medical Sciences.

### Chemicals

High Pure RNA Tissue Kit (#12033674001, Roche, Germany) was used for RNA extraction and EXPRESS One-Step SYBR® GreenER™ SuperMix Kit (#11780-200, Invitrogen, USA) for qRT-PCR. Bio-Rad Protein Assay Kit (#500-0002, Bio-Rad, USA) to determine protein contents. Imipramine hydrochloride obtained from Marham Daru, Iran. Tris–HCl, (ethylenediaminetetraacetic acid) EDTA, Sodium fluoride (NaF), sodium orthovanadate (Na_3_VO_4_), β-glycerol phosphate, sodium deoxycholate (NaDC), complete protease inhibitor cocktail (P8340), phenylmethylsulfonyl fluoride (PMSF), Sodium dodecyl sulfate (SDS), 2-mercaptoethanol (2-ME), Bromophenol blue (BPB), glycerol, and Tris Buffered Saline with Tween® 20 (TBST ), and Tween® 20 purchased from Sigma-Aldrich, Germany.

### Crocin extraction

Crocin was extracted and purified as previously described by Hadizadeh and colleagues [38]. Ten g saffron stigmas powders were suspended in 25 mL ethanol 80% (0°C) and vortexed for 2 min. After that, the suspension was centrifuged at 4000 rpm for 10 min and the supernatant was separated. This step was repeated 6 times by addition of 25 mL ethanol 80%. The resulting extract was kept in a sealed thick walled glass container at -5°C for 24 days in darkness. The formed crystals were separated from the solution and washed with acetone to remove remaining water. The obtained crystals were then dissolved in 120 mL ethanol 80% and kept at -5°C for 20 extra days. The purity of total crocin was more than 97% and the amount of obtained crocin from the initial stigmas powder was 10%. The purity of crocin crystals was determined using UV-visible spectrophotometery and HPLC [38].

### Treatments

Thirty rats were randomly divided into 5 different treatment groups (n = 6). Different doses of crocin (12.5, 25, and 50 mg/kg) [28,39] were administered intraperitoneally (IP) for 21 days. Neutral and positive control groups received (IP) 1 mL/kg saline and 10 mg/kg imipramine, respectively [40,41]. Crocin and imipramine were dissolved in saline right before injections. All treatments were injected in a volume of 1 mL/kg. After 21 days of treatment, rats were examined in the FST one hour after the final injections. Then, all treated rats were killed by decapitation. Hippocampi were separated immediately and frozen in liquid nitrogen and stored at -80°C until use.

### Forced swimming test (FST)

The FST was conducted between 10:00 and 14:00 hours. The test involved two individual sections (2 days) using a cylindrical tank made of glass, 80 cm tall, 30 cm in diameter, and filled with water (23-25°C) to a depth of 40 cm in which rats could not touch the bottom of the tank. On the 1st day (pretest), rats placed individually in the tank for 15 min and then they were removed from the water and placed in cages equipped with warmers. Tanks were cleaned and filled with fresh water between experiments. Twenty four hours after the pretest, rats were retested for 6 min under the same condition. The retest observations were recorded using a Panasonic digital camcorder (Model NO. NV-DS65EN). During the last 4 min of the retest, immobility (no additional activity other than those movements necessary to keep the rat head off the water) times were scored by an observer unaware of the treatment groups [42,43].

### Tissue collection

Rats (n = 6) immediately were sacrificed by decapitation after the FST under stress free conditions. Each decapitation performed in a room isolated from other rodents. The animal head was positioned completely in the opening of the guillotine and guillotine lever was quickly depressed. After that, Brain was removed, dissected on ice in 3–4 min following decapitation. The brain was cut in half using a midline incision and the midbrain was gently removed. The hippocampus is delineated by a large vessel running along its length. Hippocampi were isolated; tissue at each end of them was cut, washed by saline, rapidly frozen in liquid nitrogen and stored at -80°C for subsequent processing.

### Protein extraction

To prepare samples for western blotting, tissues were homogenized in the homogenization buffer containing Tris–HCl 50 mM ( pH: 7.4), 2 mM EDTA, 10 mM NaF, 1 mM Na_3_VO_4_, 10 mM β-glycerol phosphate, 0.2% w/v NaDC, 1 mM PMSF, and complete protease inhibitor cocktail using polytron homogenizer (POLYTRON® PT 10–35, Kinematica, Switzerland) in ice. After centrifugation at 10000 × g for 15 min at 4°C, Supernatants were collected on ice and protein contents were determined using Bio-Rad Protein Assay Kit and all concentrations were adjusted to 10 mg/mL. Equal volumes of SDS sample buffer containing 4% w/v SDS, 10% v/v 2-ME, 100 mM Tris-base, 0.2% w/v BPB, and 20% v/v glycerol were added to the samples and incubated in boiling water for 5 min. Blue homogenates were stored at -80°C until use.

### Western blot

Immuno blotting analysis performed on the prepared samples to assess the levels of CREB, p-CREB, BDNF, and VGF. Briefly, samples containing equivalent amounts of 50 μg of total protein were loaded to SDS-PAGE gel and then transferred to PVDF membrane by electrophoresis. Blots were blocked with 5% non-fat dry milk in TBST for 3 h at room temperature. After blocking, blots were probed with specific primary antibodies: rabbit monoclonal anti-serum against CREB (#9197, Cell Signaling, USA), mouse monoclonal anti-serum against p-CREB (Ser133) (#9196, Cell Signaling, USA), rabbit polyclonal anti-serum against BDNF (#ab46176, Abcam, USA) and VGF (#ab74140, Abcam, USA), and mouse and rabbit monoclonal anti-serums against β-actin (# 3700 and # 4970, Cell Signaling, USA) at 1:1000 dilutions for 2 h at room temperature. Membranes were washed 3 times with 0.1% Tween® 20 and TBST. Then, blots were incubated with antimouse and rabbit horse radish peroxidase labeled IgG (#7076 and #7074, Cell Signaling, USA) as secondary antibodies at 1:3000 dilutions for 1 h at room temperature. Finally, protein bands were visualized using an enhanced chemiluminescence reagent (Pierce ECL western blotting substrate) and Alliance Gel-doc (Alliance 4.7 Gel doc, UVtec UK). UV Tec software (UK) was used to semi quantify protein bands intensities. All blots were normalized against intensities of corresponding β-actin protein bands.

### RNA extraction

Total RNAs were extracted from rat hippocampi using High Pure RNA Tissue Kit according to the manufacturer’s instructions. The quantity and quality of the isolated RNAs were assessed using NanoDrop 2000 UV–vis spectrophotometer (Thermo Scientific, USA).

### Quantitative RT-PCR

QRT-PCR was performed to analyze transcript levels of CREB, BDNF, and VGF using EXPRESS One-Step SYBR® GreenER™ SuperMix Kit for one-step qRT-PCR according to the manufacturer’s instructions and a StepOne™ Real-Time PCR System (ABI, USA). Data were analyzed using the ΔΔCt method [44].

The following real-time PCR protocol was used for all genes: activation of reverse transcriptase and cDNA synthesis (5 min @ 50°C), PCR activation (2 min @ 95°C), 40 cycles of denaturation (15 s @ 95°C) and annealing/extension (1 min @ 60°C). At the end of the PCR, a melting curve analysis was performed by gradually increasing the temperature from 60 to 95°C with a heating rate of 0.3°C/s.

Primers for the selected genes were designed using Beacon designer 7.8 (Biosoft, USA) and their specificity was confirmed by BLAST (http://www.ncbi.nlm.nih.gov/tools/primer-blast/). β-actin was used as endogenous control gene. Primers were purchased from Metabion international AG, Germany (Table 1).

**Table 1:** Primers used for qRT-PCR

**Gene**			**Amplicon length (bp)**
VGF	Forward	5′-GATGACGACGACGAAGAC-3′	100
	Reverse	5′-CGATGATGCTGACCACAT-3′	
β-actin	Forward	5′GGGAAATCGTGCGTGACATT-3′	76
	Reverse	5′- GCGGCAGTGGCCATCTC-3′	
CREB	Forward	5′-CCAAACTAGCAGTGGGCAGT-3′	140
	Reverse	5′- GAATGGTAGTACCCGGCTGA-3′	
BDNF	Forward	5′-TCTACGAGACCAAGTGTAATCC-3′	152
	Reverse	5′- TATGAACCGCCAGCCAAT-3′	

### Statistical analysis

Data were analyzed using GraphPad InStat version 3.00 (GraphPad Software, San Diego, California, USA) with One-way Analysis of Variance (ANOVA) followed by Tukey post-hoc test and plotted in GraphPad Prism version 3.00 (GraphPad Software, San Diego California USA). All data presented as mean ± Standard error of the mean (S.E.M). *P* values less than 0.05 were considered to be statistically significant.

## Results

### Forced swimming test

**Figure 1:**
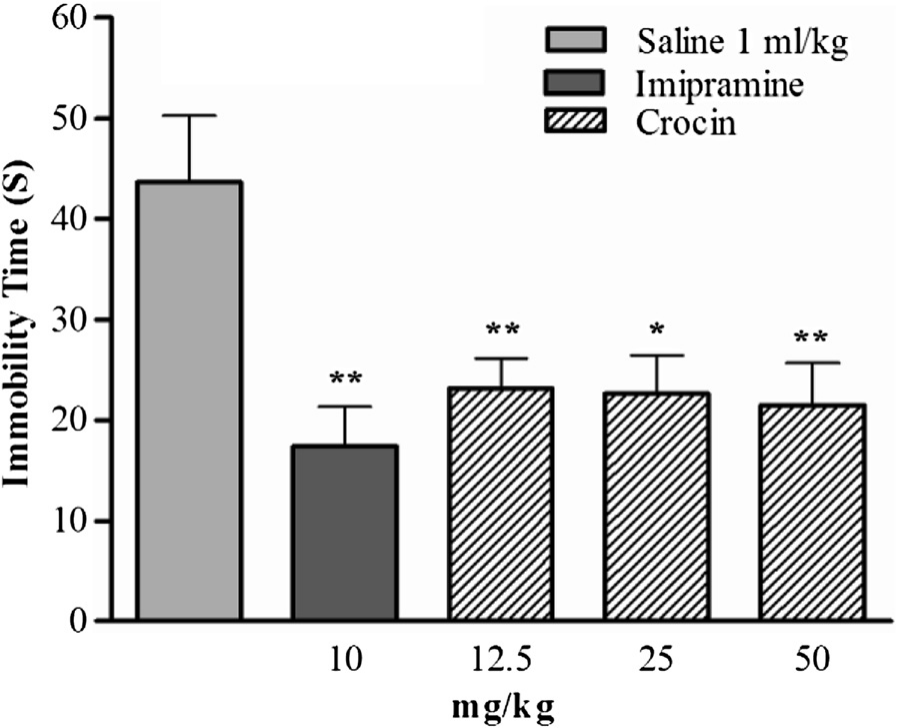
**Effects of the subacute administration of crocin and imipramine on immobility time of rats subjected to the forced swimming test.** The data are expressed as mean ± S.E.M; n = 6. All groups were compared to neutral control group (saline) according to ANOVA followed by Tukey post-hoc test: *p < 0.05, **p < 0.01.

As shown in Figure 1, subacute administration of crocin (12.5 and 50 mg/kg: **p < 0.01; 25 mg/kg: *p < 0.05) and imipramine (10 mg/kg, **p < 0.01) significantly reduced the immobility time as compared with neutral control group that received saline. All doses of crocin could reduce the immobility time, but not in a dose dependent manner.

### Western blot assay

**Figure 2:**
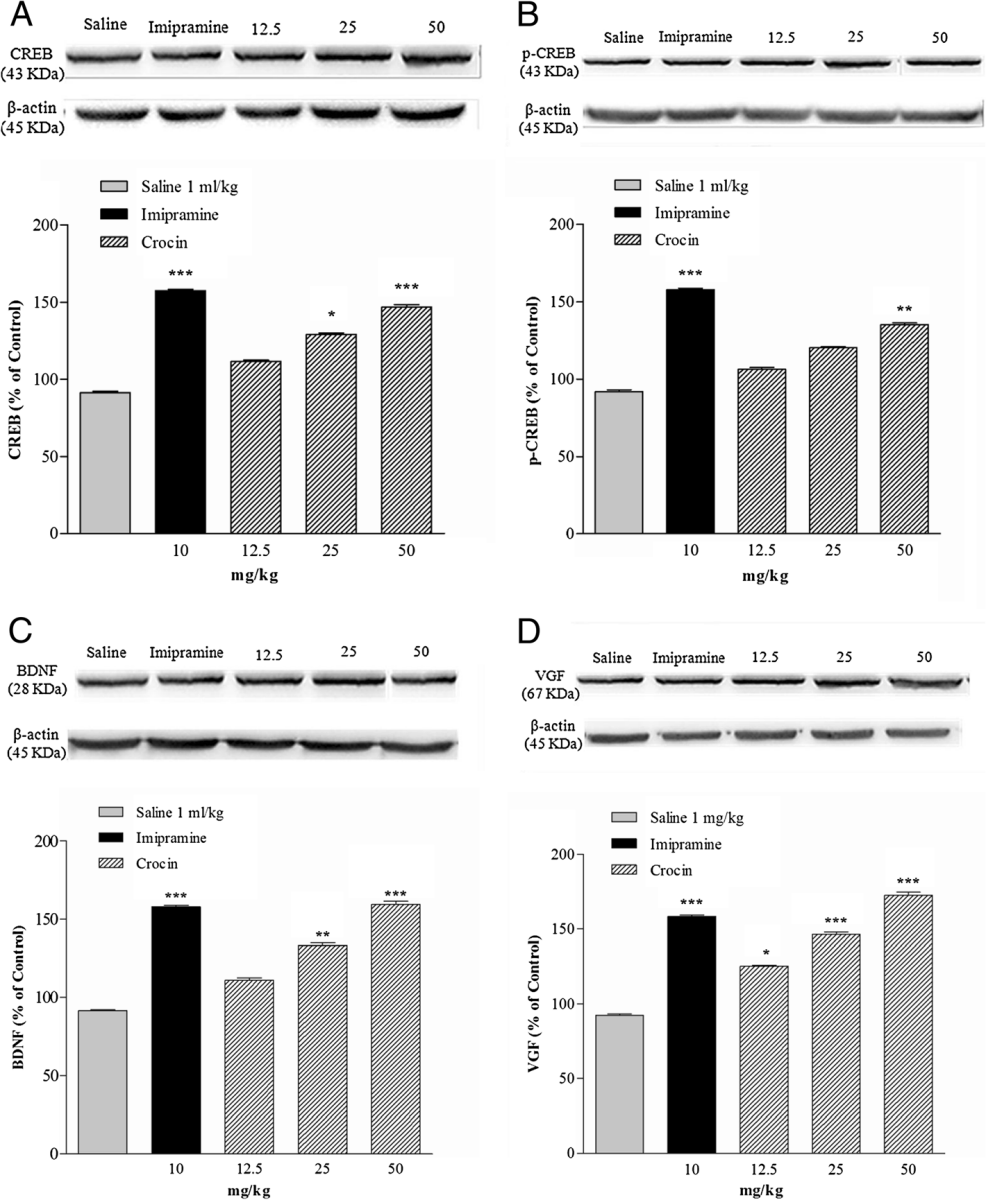
**Effects of the subacute administration of crocin and imipramine on protein levels of A: CREB, B: p-CREB, C: BDNF, and D: VGF in the hippocampi.** The graphics show the mean ± S.E.M. of separate experiments: n = 6. All groups were compared to neutral control group (saline) according to ANOVA followed by Tukey post-hoc test: *p < 0.05, **p < 0.01, ***p < 0.001. β-actin: endogenous control.

Effects of the subacute treatment with crocin (12.5, 25, and 50 mg/kg, IP) on the CREB, p-CREB, BDNF, and VGF protein expression in the hippocampus are shown in Figure 2. Statistical analysis indicated a significant and dose-dependent effect of treatment with crocin on the CREB expression as compared with control: 25 mg/kg (37.80%, *p < 0.05) and 50 mg/kg (55.6%, ***p < 0.001). Effect of imipramine on the CREB level was higher than that of in crocin treated groups (Figure 2A). Crocin at a dose of 12.5 mg/kg showed no appreciable effect on the CREB expression as compared with saline group. Treatment with high dose of crocin (50 mg/kg) significantly increased the expression of p-CREB (43.52%, **p < 0.01) as shown in Figure 2B. As shown in Figure 2C, there were 41.63% (**p < 0.01) and 67.79% (***p < 0.001) increase in the levels of BDNF after treatment with 25 and 50 mg/kg crocin as compared with control, respectively. Treatment with 12.5 mg/kg crocin could not significantly change the BDNF protein level. The effect of 50 mg/kg crocin on the level of BDNF was similar to 10 mg/kg imipramine (***p < 0.001 vs. saline). Crocin at all doses, compared to saline, could significantly and dose dependently increase the VGF levels in a dose-dependent manner: 12.5 mg/kg (32.95%, p < 0.05), 25 mg/kg (54.35%, *p* < 0.001), and 50 mg/kg (80.54%, *p* < 0.001). Crocin at doses of 25 and 50 mg/kg could increase the VGF levels similar to that of imipramine (***p < 0.001 vs. saline, Figure 2D).

### Quantitative RT-PCR

**Figure 3:**
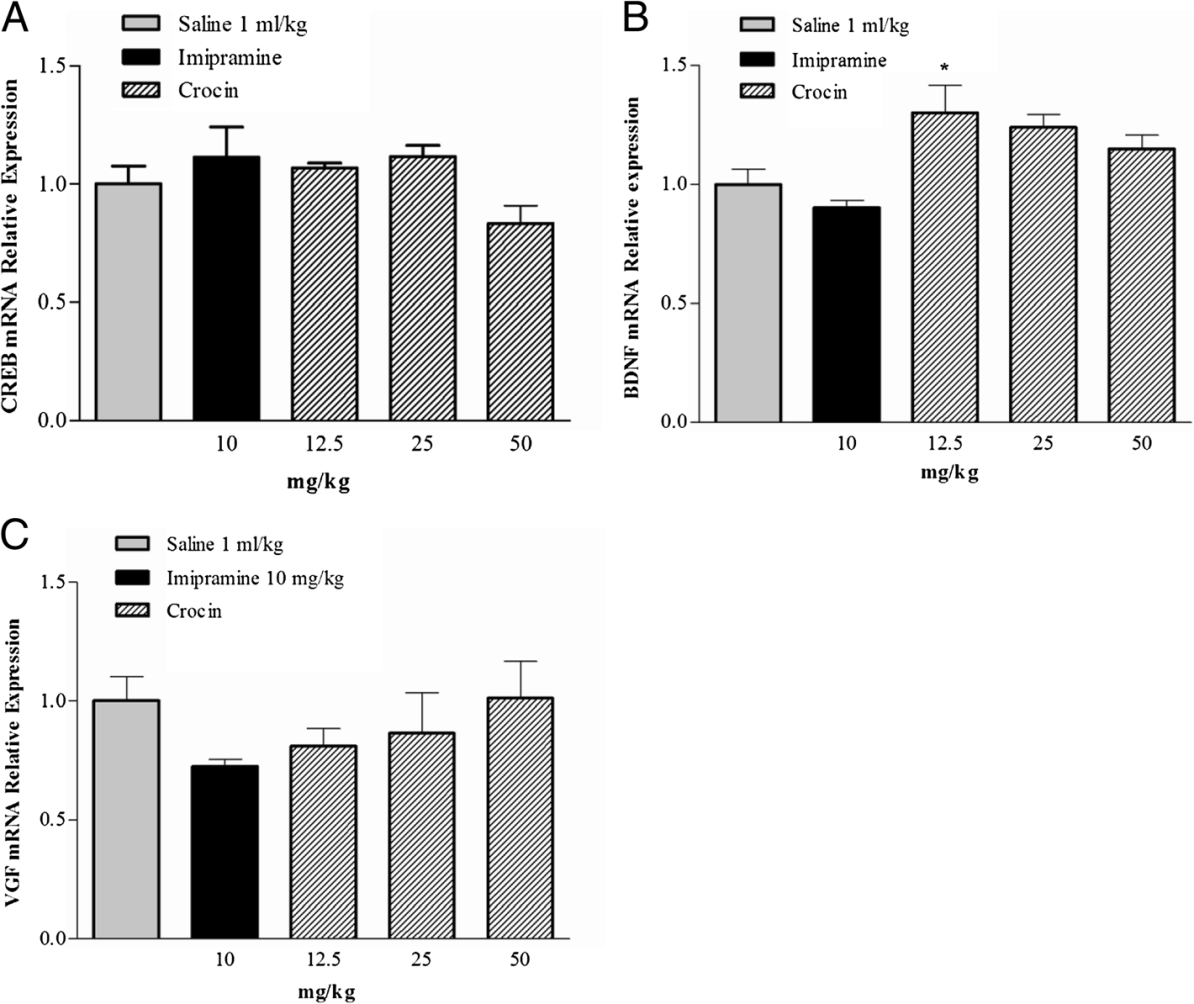
**Effects of the subacute administration of crocin and imipramine on transcript levels of A: CREB, B: BDNF, and C: VGF.** Data are expressed as mean ± S.E.M; n = 4. All groups were compared to neutral control group (saline) according to ANOVA followed by Tukey post-hoc test: *p < 0.05.

Figure 3 illustrates the effects of the subacute treatment with crocin (12.5, 25, and 50 mg/kg, IP) and imipramine (10 mg/kg, IP) on the CREB, BDNF and VGF transcript levels in rat hippocampi. The lowest dose of crocin (12.5 mg/kg) could significantly increase the BDNF transcript levels in the hippocampus (*p < 0.05) as compared with saline group. No significant changes were observed in the transcript levels of CREB and VGF in different experimental groups.

## Discussion

In the present study we demonstrated that subacute administration of crocin in all doses (12.5, 25, and 50 mg/kg) decreased the immobility time of rats in the forced swimming test (FST), however this effect was not in a dose-dependent manner. Subacute treatment with 10 mg/kg imipramine as positive control also decreased the immobility time. In order to understand the molecular mechanism of crocin-induced subacute antidepressant effects in the hippocampus, the protein and transcript levels of anti-depressant related genes were studied. Our data showed that crocin increased the protein levels of CREB, p-CREB, BDNF, and VGF in the hippocampus. Treatment with 12.5 mg/kg crocin significantly increased in BDNF transcript level.

In late 1970s, FST was accepted as a beneficial model to predict the antidepressant effects of drugs on animal [42]. The antidepressant effects of different saffron extracts and crocin in acute administration in mice and rats have already been reported in previous studies using the FST. Furthermore, in the open field activity test, crocin did not show a significant effect on total locomotion [34-37]. The studies have been reported that acute and chronic administration of imipramine (10 mg/kg) significantly reduced the immobility time and confirmed its antidepressant effects [40,41]. In our study, imipramine (10 mg/kg) like previous reports decreased the immobility time as compared with neutral control group. The administration of crocin in different doses also reduced the immobility time, similar to that of imipramine. Therefore, our results suggest that crocin has antidepressant effects in subacute treatment.

Hippocampus is a region in the brain that plays a central role in processing of emotions and controlling of behavior in response to fear and anxiety [45]. Preclinical and Clinical studies have shown that the hippocampus is affected by stress. Death and atrophy of hippocampal neurons have been reported in rats exposed to stress and high levels of glucocorticoids [46]. The reduction of hippocampus size in patients with recurrent depression and posttraumatic stress disorder has been observed [47-49]. It is well established that structural and functional modifications of hippocampus are associated with antidepressant treatments. These changes include alterations in synaptic plasticity, neurogenesis, and synaptogenesis and most likely require the transcription and protein expression of new molecules such as CREB, BDNF and VGF [7].

Chronic treatment with antidepressants enhances, activates and induces the phosphorylation of CREB that produces antidepressant behavioral response in rodents and human [7,11,50,51]. Various antidepressants have shown different effects on CREB protein and mRNA levels. For example, 21 days administration of several different types of antidepressant drugs including fluoxetine (a serotonin (5-HT) selective reuptake inhibitor), desipramine (a selective norepinephrine (NE) reuptake inhibitor), imipramine (a nonselective 5-HT and NE reuptake inhibitor, 15 mg/kg), and tranylcypromine (a monoamine oxidase inhibitor) significantly increased levels of CREB mRNA in rat hippocampus and only fluoxetine significantly increased CREB protein levels [11,51]. Conversely, similar doses and time course of administration showed that neither desmethylimipramine nor fluoxetine could increase CREB protein levels; however, both could increase CREB phosphorylation, but only in the frontal cortex and not in the hippocampus [11]. In addition, it was shown that imipramine at dose of 20 mg/kg could only increase CREB protein levels in prefrontal cortex but not in hippocampus [52]. The administration of fluoxetine and reboxetine (a NE reuptake inhibitor) for 14 days at a dose of 10 mg/kg resulted in increased levels of CREB mRNA in the hippocampus, but desipramine at the same dose did not have significant effect on CREB mRNA level [50]. In transgenic mice model of depression imipramine (10 mg/kg) increased CREB mRNA levels only in the cortex, whereas fluoxetine (10 mg/kg) could increase the levels of CREB mRNA in the cortex and the hippocampus [53]. The results of the present study showed that crocin administration could increase the CREB protein levels in the hippocampus dose dependently. P-CREB significantly increased only with 50 mg/kg of crocin. Desmethylimipramine and fluoxetine has been reported to increase CREB phosphorylation in other region of the brains like cortex [11]. In our study, no significant changes in CREB mRNA levels were observed in hippocampus. Therefore, crocin-induced changes in CREB and p-CREB protein and CREB mRNA levels may be involved in other brain regions such as cortex similar to previous reports [11,53]. However more supportive data are necessary to confirm this hypothesis.

BDNF structurally belongs to the neurotrophin family that plays an important role in regulation of neuronal differentiation including neurotransmitter content and neuronal survival [54]. Recent studies have shown that after treatment with antidepressants, levels of BDNF significantly increased in plasma [55,56]. It was evidenced that use of imipramine as a nonselective 5-HT and NE reuptake inhibitor at doses of 10 and 20 mg/kg was effective to increase BDNF protein levels in both prefrontal cortex and hippocampus [52]. It has been shown that longer treatment with citalopram (a serotonergic agent) could significantly increase the level of BDNF transcript [57]. In addition, both acute and chronic use of norepinephrine re-uptake inhibitors (desipramine and maprotiline) had no effect on BDNF mRNA levels, while serotonergic antidepressants (fluoxetine and paroxetine) altered BDNF gene expression, but not in acute administration [58]. In the current study, subacute treatment with crocin in a dose-dependent manner increased the BDNF protein levels compared to the neutral control treatment group. Crocin could significantly increase the BDNF transcript level as compared to saline group. Due to the result of present and past studies, the effect of crocin on BDNF expression levels is similar to serotonergic drugs.

VGF is a neuropeptide that has been shown to be involved in maintaining energy balance, mediating hippocampal synaptic plasticity, and antidepressant responses [7,59]. In several animal models of depression local application of VGF into the midbrain or hippocampus produced antidepressant responses [60]. Due to the different studies, VGF gene is an important target for BDNF and serotonin. This agent besides exercise may activate intracellular pathways that may lead to the VGF expression [59]. Antidepressants do not show the same effects on VGF gene expression. Although Hunsberger and colleagues reported that VGF expression was not affected by different classes of antidepressants [61], there are some reports that show fluoxetine and paroxetine, but not imipramine and desipramine could increase the VGF expression [60]. Our results showed that transcript levels of VGF were not increased following administration of different doses of crocin, however, VGF protein expression significantly and dose-dependently elevated after treatment with crocin.

## Conclusions

In conclusion, our study showed that subacute administration of crocin has antidepressant effects in rats. Crocin administration significantly increased the CREB, p-CREB, BDNF, and VGF protein expressions in rat hippocampus.

## Competing interests

The authors declare that they have no competing interests.

## Authors’ contributions

HH and KA designed the study. SM and BMR were the supervisors. FVH and VN participated in doing the experiments. FVH draft the manuscript. All authors read and approved the final manuscript.
